# Canagliflozin alleviates acetaminophen-induced renal and hepatic injury in mice by modulating the *p-*GSK3β/Fyn-kinase/Nrf-2 and *p-*AMPK-α/STAT-3/SOCS-3 pathways

**DOI:** 10.1038/s41598-024-82163-7

**Published:** 2025-01-03

**Authors:** Abeer Bishr, Bassant M. El-Mokadem, Asmaa A. Gomaa

**Affiliations:** 1 Department of Pharmacology and Toxicology, Faculty of Pharmacy, Ahram Canadian University, Giza, Egypt; 2 Department of Pharmacology and Toxicology, Faculty of Pharmacy, Egyptian Chinese University, Cairo, Egypt

**Keywords:** Acetaminophen, Canagliflozin, Nephrotoxicity, Hepatotoxicity, *p-*AMPK-α, Fyn-kinase, Biomarkers, Nephrology

## Abstract

Despite the fact that canagliflozin (Cana), a sodium-glucose cotransporter 2 inhibitor, is an anti-diabetic medication with additional effects on the kidney, there is limited experimental data to deliberate its hepato-reno-protective potentiality. Acetaminophen (APAP) overdose remains one of the prominent contributors to hepato-renal damage. Aim: Our study assessed the novel effect of Cana against APAP-induced toxicities. Main methods: mice were randomized into five groups: negative control, Cana_25_, APAP, Cana_10_ + APAP, and Cana_25_ + APAP. Cana was given for 5 days; a single dose of APAP was injected on the 6^th^ day, followed by the scarification of animals 24 h later. Key findings: Pre-treatment with Cana ameliorated hepatic and renal functions, whereas, on the molecular levels, Cana promoted hepatic/renal *P*-AMP-activated protein kinase-α/ protein kinase B (*p*-Akt)/Glycogen synthase kinase (*p*-GSK3β) protein expression. Alternatively, Cana dampened the expression of STAT-3 and Fyn-kinase genes with a subsequent increase in the contents of suppressor of cytokine signaling (SOCS)-3 and also boosted the contents of the nuclear factor erythroid related factor 2 (Nrf-2)/heme oxygenase (HO)-1/ NADPH quinone oxidoreductase (NQO)-1 axis. The crosstalk between these paths ameliorated the APAP-induced hepatorenal structural alterations. Significance: Cana hepatorenal protective impact was provoked partly through modulating *p-*AMPK-α /SOCS-3/STAT-3 and GSK3β/Fyn-kinase signaling for its anti-inflammatory and antioxidant effects.

## Introduction

Drug-induced hepatorenal injury has emerged as the leading cause of acute hepatic and renal impairment^1^. Acetaminophen (APAP) has been extensively and safely utilized for decades as a pain reliever and antipyretic at recommended doses^1^; however, overdose ingestion leads to liver and renal damage^2^. At higher doses of APAP, the Cyp450 system transforms N-acetyl-p-benzoquinone imine (NAPQI), a reactive hazardous intermediate metabolite. NAPQI conjugates glutathione (GSH), leading to GSH depletion^3^. This leads to oxidative stress (OS) and subsequent cellular damage in hepatocytes and renal cells with mitochondrial dysfunction elevation of reactive oxygen species (ROS)^4,5^, promoting necrosis and inflammation via pathways like JAK-2/STAT-3^6^. On the contrary, the cellular damage cause failure of Nrf-2 pathway that further impairs antioxidant defenses in both liver and kidney tissues^7,8^. The combined effect of ROS accumulation, GSH depletion, and inflammation drives the pathogenesis of APAP-induced organ toxicity that may ends with hepatic and renal cell death^9^.

Hepatic and renal diseases are among the most debilitating ailments; this is why it is imperative to choose a suitable protective medication. Sodium-glucose cotransporter 2 (SGLT2) inhibitors, used as antidiabetic drugs, have demonstrated efficacy in reducing renal disease progression and liver-related outcomes in diabetic patients^10,11^. Despite scanty of experimental data on SGLT2 inhibitors’ effects on hepato/renal injury models, Hasan et al.^12^ and Abdelrahman et al.^13^ have found exceptional antioxidant and anti-inflammatory properties in isoprenaline- and cisplatin-induced renal damage. However, Cana didn’t show any potential to protect the kidneys in cases of oxalate-induced nephrocalcinosis^14^ or in the rat remnant kidney model of chronic kidney disease^15^.

AMP-activated protein kinase (*p-*AMPK-α), an intracellular energy sensor and regulator of mitochondrial and cellular hemostasis, is one of the key indicators of low energy status after APAP poisoning^16,17^. According to existing literature, when p-AMPK-α is activated, its downstream cues are also activated, such as protein kinase B (*p*-Akt), that have a vital role in attenuating APAP-induced toxicities^18^. This cascade culminates in the activation of nuclear factor erythroid 2-related factor 2 (Nrf-2) through the Glycogen synthase kinase-3β (GSK3β)/Fyn-Kinase axis, as suggested by Fan et al.^19^ and Lee et al.^20^. Protein kinase B (Akt), a serine/threonine protein kinase, phosphorylates and inactivates Glycogen Synthase Kinase-3 Beta (GSK3β)^21^. GSK3β is an upstream regulator of Fyn-kinase and has a negative impact on Nrf2 stability^22^.

Despite the significant pharmacological effects of Cana, its efficacy in countering APAP-induced hepatic and nephrotoxicity remains uncertain. Accordingly, we aimed to highlight Cana’s capability to mitigate APAP-induced liver and kidney damage and to reveal additional mechanisms involved in its potential effect.

## Materials & methods

### Drug and chemicals

In the present experiment, APAP was procured from Sigma Aldrich Chemicals (St. Louis, MO, USA), and Cana was acquired from Janssen Pharmaceuticals (Inc., UK).

### Animals

Male ICR mice were procured from the Ahram Canadian University Faculty of Pharmacy’s animal house (ACU; Giza, Egypt). Mice were housed in a temperature- and humidity-controlled environment (24°C, 60 ± 10% humidity with a 12-h light/12-h dark cycle) and free access to water and food. The experimental procedures were approved by the research ethical committee, Faculty of Pharmacy, Ahram Canadian University (REC 1823) that adheres to the National Institute of Health (NIH) for Care and Use of Laboratory Animals (NIH, No. 85–23, revised 1996).

### Experimental design

In the present study, the mice were randomly assigned to the following groups (n = 6): negative control (0.5% hydroxypropyl methylcellulose), Cana_25_ (25mg/kg), APAP (400mg/kg), APAP + Cana_10_ (10mg/kg), and APAP + Cana_25_ (25mg/kg). APAP (400mg/kg) was administered intraperitoneally to induce liver and renal damage. Before APAP challenge, the mice were administered with Cana (10, 25mg/kg) for 5 days. The mice were euthanized 24 h following the APAP treatment, and blood samples were collected. The dose of Cana (10 mg/kg) was based on earlier study that reported the protective effect of Cana-against cisplatin induced nephrotoxicity^23^, moreover Cana administration with a dose 25 mg/kg was according to Sun et al.^24^. On the other hand, dose of acetaminophen depended on the previous study documented by Feng et al.^25^.

### Sampling

After animals were anesthetized with ketamine (75 mg/kg) and xylazine (5 mg/kg) interperitoneally, blood samples were collected from the retro-orbital sinuses of each animal, and the obtained serum was stored at -80 °C, pending assessment of renal and liver functions, thereafter all animals were sacrificed by cervical dislocation. Animals’ kidneys (left and right) and livers after divided into two portions were harvested and washed with ice-cold normal saline. Left kidney tissues and a 1^st^ portion of liver for (6 mice/group) were homogenized in cold phosphate-buffered saline and centrifuged under adjusted conditions (10,000 × *g* for 20 min at 4 °C), and the resultant supernatant was used for enzyme-linked immunosorbent assays** (**ELISA). Simultaneously, the animal’s right kidney and 2^nd^ portion of liver (3 mice/group) were used for real-time polymerase chain reaction (qRT-PCR) and western blot analyses, and the remaining 3 right kidneys and other liver tissues (3 mice/group) were immersed in 10% neutral buffered formalin for 72 h and processed for histopathological and immune-histochemical (IHC) investigations.

### Biochemical analysis

#### Evaluation of liver and kidney functions and GSH content calorimetrically

Serum levels of blood urea nitrogen (BUN) and creatinine were determined using the colorimetric kits QuantiChrom™ Urea Assay (Cat. # DIUR-500) and QuantiChrom™ Creatinine assay (Cat. # DICT-500) kits, respectively, were procured from (Hayward, CA, USA). While serum levels of aspartate (AST) and alanine transaminase (ALT) were assessed using colorimetric kits (Teco Diagnostics, CA, USA; Cat. # A559–150, Cat. # A524–150, respectively) in line with the manufacturer’s instructions. Moreover, GSH contents in liver and renal tissues were spectrophotometrically measured using commercial kits supplied by Biodiagnostic (Cat. # GR 25 11; Giza, Egypt) according to the methods of Beutler et al.^26^.

#### Evaluation of NGAL, KIM-1, HO-1 SOC-3, and NQO-1 using ELISA technique

The kidney contents of neutrophil gelatinase-associated lipocalin (NGAL; Cat. # MBS260195), kidney injury molecule-1 (KIM-1; Cat. # MBS355395), and hepatic/renal contents of hemoxgenase-1 (HO-1; Cat. # MBS9303006) were evaluated using the ELISA assay kits purchased from MyBiosource, CA, USA. Renal and liver homogenates were also used to assess the following parameters using the corresponding ELISA kit; suppressor of cytokine signaling-3 (SOCS-3; Cloud-Clone Corp, TX, USA; Cat. # SEB684Ra) and NAD(P)H quinone dehydrogenase 1 (NQO-1, LifeSpan Biosciences, WA, USA; Cat. # LS-F32218) according to the manufacturer’s instructions.

#### Quantitative real time PCR (qRT-PCR)

Direct-zol RNA Miniprep Plus (Cat. # R2072, Zymo Research Corp, CA, USA) was used to isolate total RNA from tissue lysate, and then RNA was quantified and graded using a Beckman dual spectrophotometer (USA). The extracted RNA was reverse-transcribed, and then PCR was performed via the Superscript IV One-Step RT-PCR kit (Thermo Fisher Scientific, MA, USA; Cat. # 12,594,100), using the following program: incubation at 94°C for 15 min, then 94°C for 15 s (40 cycles), followed by 60°C for 30 s, and 70°C for 30 s in a qPCR analyzer (QIAGEN, Germany). The sequences of primers are shown in Table 1. The mRNA expression of signal transducer and activator transcription 3 (STAT-3) and Fyn-kinase utilizing ß-Actin as a housekeeping gene was calculated using 2 ^– (ΔΔ Ct)^ method.

**Table 1:** Sequences of the primers for quantitative real-time PCR.

	**Forward sequence**	Reverse sequence
STAT-3	5’-CAGCAATACCATTGACCTGCC-3’	5’-TTTGGCTGCTTAAGGGGTGG-3’
Fyn-kinase	5’-GACTCTTAAACCAGGACA-3’	5’-CAGGTTTTCACCAGGTTG-3’
β-actin	5’-CCCATCTATGAGGGTTACGC-3’	5’-TTTAATGTCACGCACGATTTC-3’

#### Determination of p-AMPK-α, p-Akt, and p-GSK3β via Western blot analysis

Regarding western blot parameters, liver and kidney samples were homogenized in radio-immunoprecipitation assay (RIPA) buffer (150 mM NaCl, 0.1% Triton X-100, 0.5% sodium deoxycholate, 0.1% sodium dodecyl sulfate and 50 mM Tris–HCl pH 8.0; Bio Basic, Markham, Ontario, Canada; Cat. #: PL005-5X10ML) with protease inhibitor using a stainless-steel homogenizer (Heidolph Diax 900, Germany). ReadyPrep™ protein extraction kit (Bio-Rad; CA, USA; Cat. # 163–2086) was used for total protein extraction from renal and hepatic tissues according to the manufacturers instructions. 30–50 μg protein samples were estimated using the Bradford Protein Assay Kit (Bio Basic, Ontario, Canada, Cat. # SK3041). Afterward, polyacrylamide gel electrophoresis (SDS-PAGE) was used to separate protein aliquots by molecular weight and transfer nitrocellulose membranes (Bio-Rad Labs, CA, USA). The membrane is then blocked with tris-buffered saline with Tween 20 (TBST) and 3% bovine serum albumin (BSA) for 1 h at room temperature. In order to detect phosphorylated forms of AMP-activated protein kinase (*P*Thr172-AMPK-α, Sigma. MO, USA; Cat. # SAB4503754), protein kinase B (*p* S473-Akt, Cell signaling technology, MA, USA; Cat. # 4051), and Glycogen Synthase Kinase-3 Beta phosphorylated at serine 9 (*P* S9-GSK3β Abcam, CB, UK; Cat. # ab75814) primary antibodies were purchased and incubated at 4°C overnight with membranes. The membranes were treated with horseradish peroxidase (HRP)-labelled secondary antibody (Bio-Rad Labs, CA, USA; Cat. # 1,706,431). The chemiluminescence detection was performed via the Amersham® detection kit (Cytiva, MA, USA) according to the manufacturer’s instructions, and then the protein level of each sample was quantified by utilizing the densitometric analysis with Bio-Rad software (Bio Rad, CA, USA) using a scanning laser densitometer (Biomed® Instruments, CA, USA). Results were obtained after normalization to housekeeping protein; β-actin.

### Histopathological examinations

Formalin-fixed liver and kidney specimens were dehydrated with graded concentrations of ethanol, cleared in xylene, infiltrated with Paraplast synthetic wax, and then embedded into tissue blocks*.* Latterly via rotatory microtome, the paraffin-embedded samples were cut into 5 μm sections for full parenchyma demonstration in different samples then mounted on glass slides stained with Hematoxylin & Eosin (H & E) for blinded light microscopic examination (Olympus BX50, Tokyo, Japan) by experienced histologists under high power magnification (× 400; scale bar 50 μm)^27^.

### Immunohistochemical staining of Nrf-2

Briefly, paraffin-embedded kidney and liver sections were deparaffinized, deepened in 3% H_2_O_2_ in methanol, and incubated for 15 min at room temperature with the Nrf-2 primary monoclonal antibody procured from Gene Tex, CA, USA (1:1000, GTX103322) for 1 h at 37^○^C. Thereafter, tissue slices were washed three times with phosphate buffer saline (PBS) and incubated with HRP secondary antibody (Abcam, CB, UK; Cat. # ab64264) for 60 min at 25⁰C. Nrf-2 immune reactivity was indicated in each section by incubation with diaminobenzidine (DAB) solution (Abcam, CB, UK; Cat. # ab64238) and counterstained with hematoxylin for microscopical examination. Four random non-overlapping fields per tissue section from each sample were analyzed for quantification of the % area of Nrf-2 expression in immuno-stained sections with blinded investigators to the groups. Data were developed through a full-HD microscopic camera operated by a Leica application unit customized for tissue analysis (Leica Microsystems GmbH, Germany).

### Statistical analysis

All data were represented as means ± standard deviation (SD), and statistical evaluation was performed using one-way analysis of variance (ANOVA) and Tukey’s post-hoc test for multiple comparisons. GraphPad Prism 6 software (GraphPad Software, CA, USA) was used to carry out the statistical analysis, and *p* < 0.05 was set as statistical significance.

## Results

Since the data obtained from the Cana_25_ group demonstrated no considerable difference compared to those obtained from the negative control group, all comparisons among groups were performed against the negative control group.

### Canagliflozin improves hepatic functions in APAP-treated mice

**Fig. 1:**
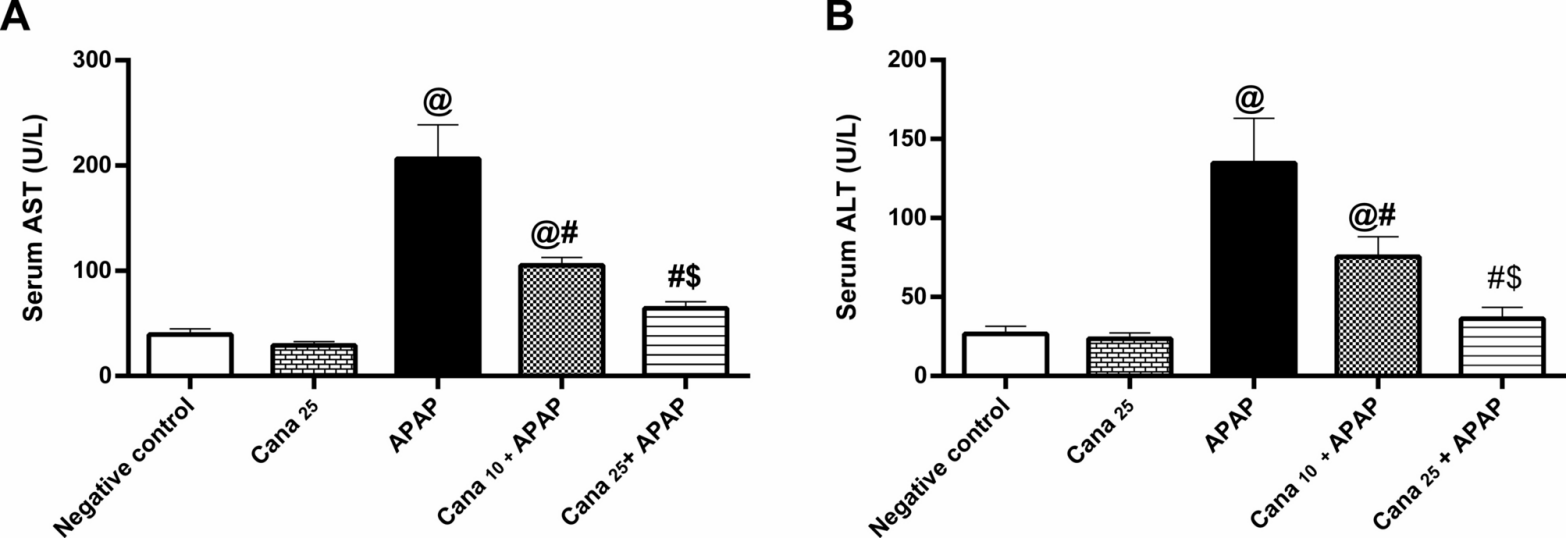
Effect of canagliflozin pre-treatment on serum **(A)** AST and **(B)** ALT levels on acetaminophen-treated mice. Data are presented as mean ± SD (n = 6) and were analyzed using one-way ANOVA followed by Tukey’s post hoc test. P < 0.05, as compared to the (@) Negative control, (#) APAP, and ($) Cana_10_ + APAP groups. APAP: acetaminophen; ALT: alanine aminotransferase; AST: aspartate aminotransferase; Cana_10_: 10 mg/kg canagliflozin: Cana_25_: 25 mg/kg canagliflozin.

The obtained results from Fig. 1 revealed that (A) AST and (B) ALT serum levels were dramatically elevated in mice treated with APAP to reach 524.6% and 505%, respectively, as compared to the negative control animals. However, pre-treatment with Cana in doses (10 and 25 mg/kg) significantly hampered APAP-induced ALT and AST elevations. Of note, Cana_25_ overrides Cana_10_.

### Canagliflozin averts APAP-induced renal dysfunction

**Fig. 2:**
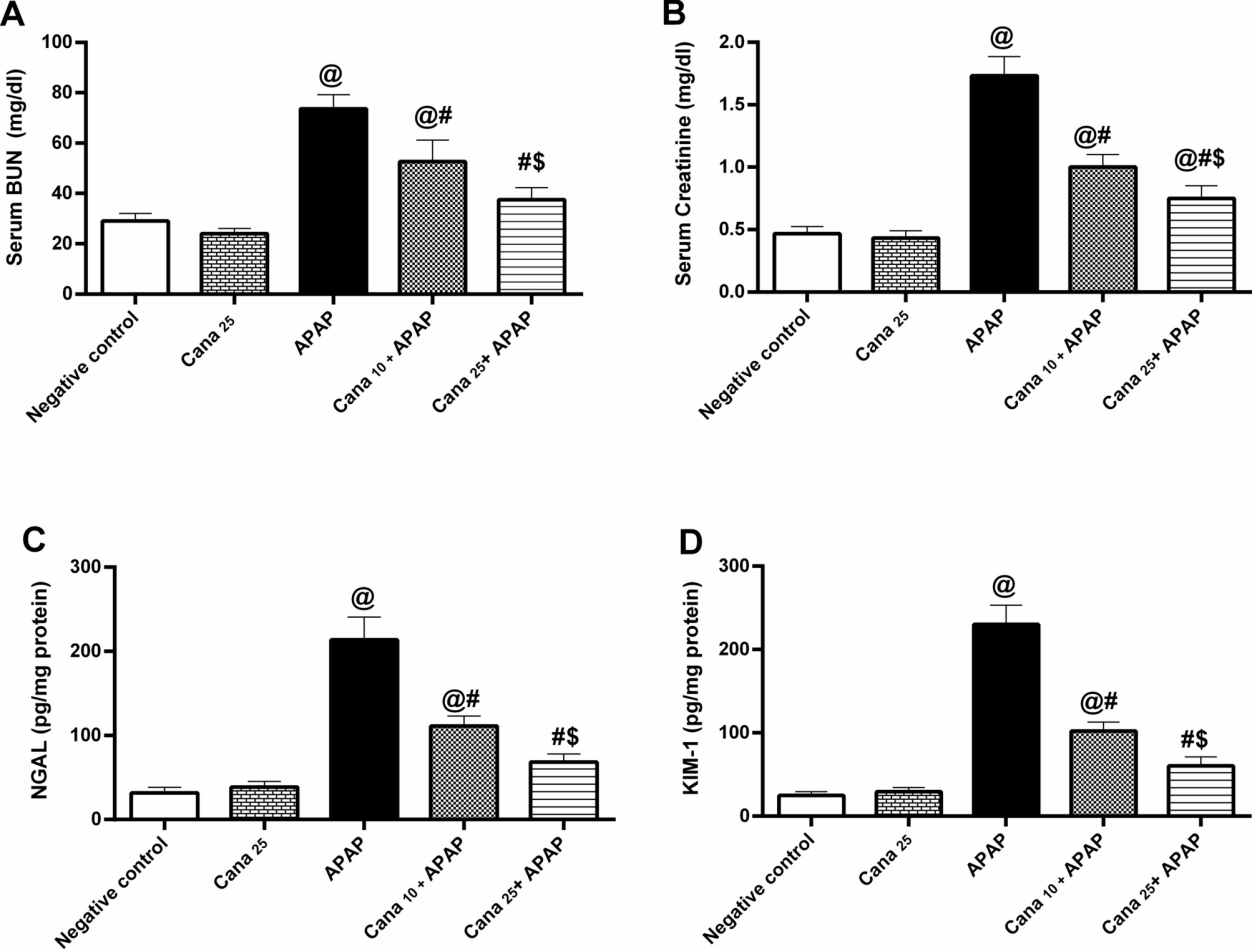
Effect of canagliflozin pre-treatment on serum **(A)** BUN and **(B)** creatinine levels, as well as **(C)** NGAL and **(D)** KIM-1 contents on acetaminophen-treated mice. Data are presented as mean ± SD (n = 6) and were analyzed using one-way ANOVA followed by Tukey’s *post-hoc* test. P < 0.05, as compared to the (@) Negative control, (#) APAP, and ($) Cana_10_ + APAP groups. APAP: acetaminophen; BUN: blood urinary nitrogen; Cana_10_: 10 mg/kg canagliflozin; Cana_25_: 25 mg/kg canagliflozin; KIM-1: kidney injury molecule-1; NGAL: neutrophil gelatinase-associated lipocalin.

As illustrated in Fig. 2, APAP administration to mice triggered a significant upsurge in (A) BUN, (B) creatinine, (C) NGAL, and (D) KIM-1 serum levels by 2.5, 3.7, 6.7, and 9.2-folds, respectively, compared to their baseline values. In contrast, the administration of Cana in a dose-dependent manner (10 mg/kg and 25 mg/kg) significantly diminished the increment of kidney function tests and tubular injury markers NGAL, as well as, KIM-1, compared to the APAP group, verifying Cana’s beneficial effect in rescuing cells from APAP-induced kidney injury.

### Canagliflozin modulates p-Akt/ p-GSK3β/Fyn-kinase cue in APAP- treated mice

**Fig. 3:**
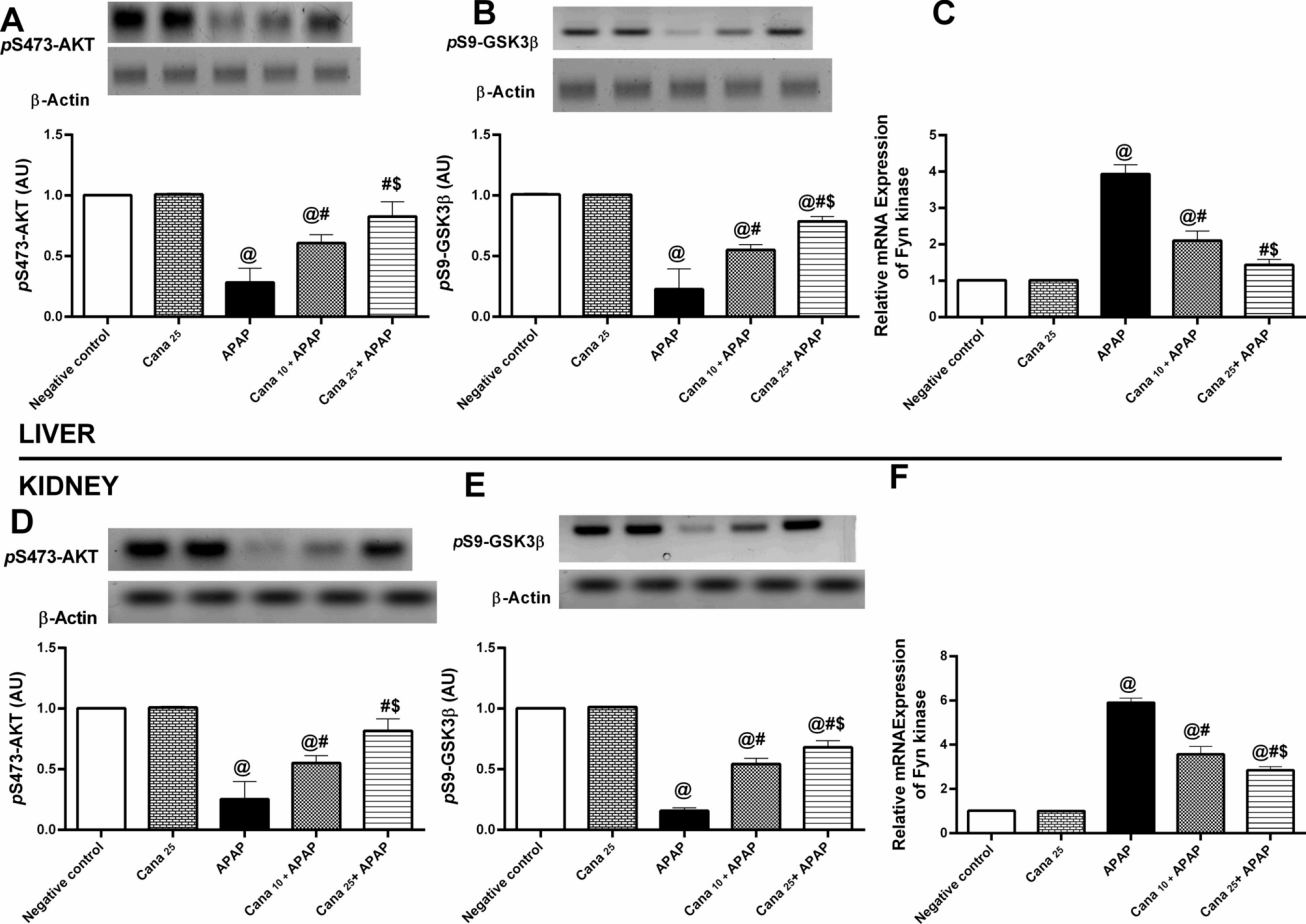
Effect of canagliflozin pre-treatment on hepatic/renal **(A, D)** *p* S473-Akt and **(B, E)** *P* S9-GSK3β protein expression, and **(C, F)** Fyn-kinase gene expression on acetaminophen-treated mice. Data are presented as mean ± SD (n = 3) and were analyzed using one-way ANOVA followed by Tukey’s *post-hoc* test. P < 0.05, as compared to the (@) Negative control, (#) APAP, and ($) Cana_10_ + APAP groups. APAP: acetaminophen; AU: arbitrary unit; Cana_10_: 10 mg/kg canagliflozin; Cana_25_: 25 mg/kg canagliflozin;* P* S9-GSK3β*:* Glycogen Synthase Kinase-3 Beta phosphorylated at serine 9;* p* S473-Akt: protein kinase B phosphorylated at serine 473. Original blots are presented in supplementary Fig. 1.

Regarding normal animals, APAP-in-toxified mice provoked a significant dampening in hepatic and renal protein expression of (A, D) *p* S473-Akt by (72% & 75%), and (B, E) *P* S9-GSK3β by (78% & 84%) respectively, which consequently raised the gene expression of (C, F) Fyn-kinase, as shown in Fig. 3. Looking at the Cana-treated groups, it was shown that 10 and 25 mg/kg Cana administration markedly reversed APAP changes on *p*-Akt/ *p-*GSK3β/Fyn-kinase axis. Surprisingly, Cana_25_ has a superior beneficial effect relative to Cana_10_.

### Canagliflozin augments the Nrf-2/HO-1/NQO-1/GSH cue in APAP treated mice

**Fig. 4:**
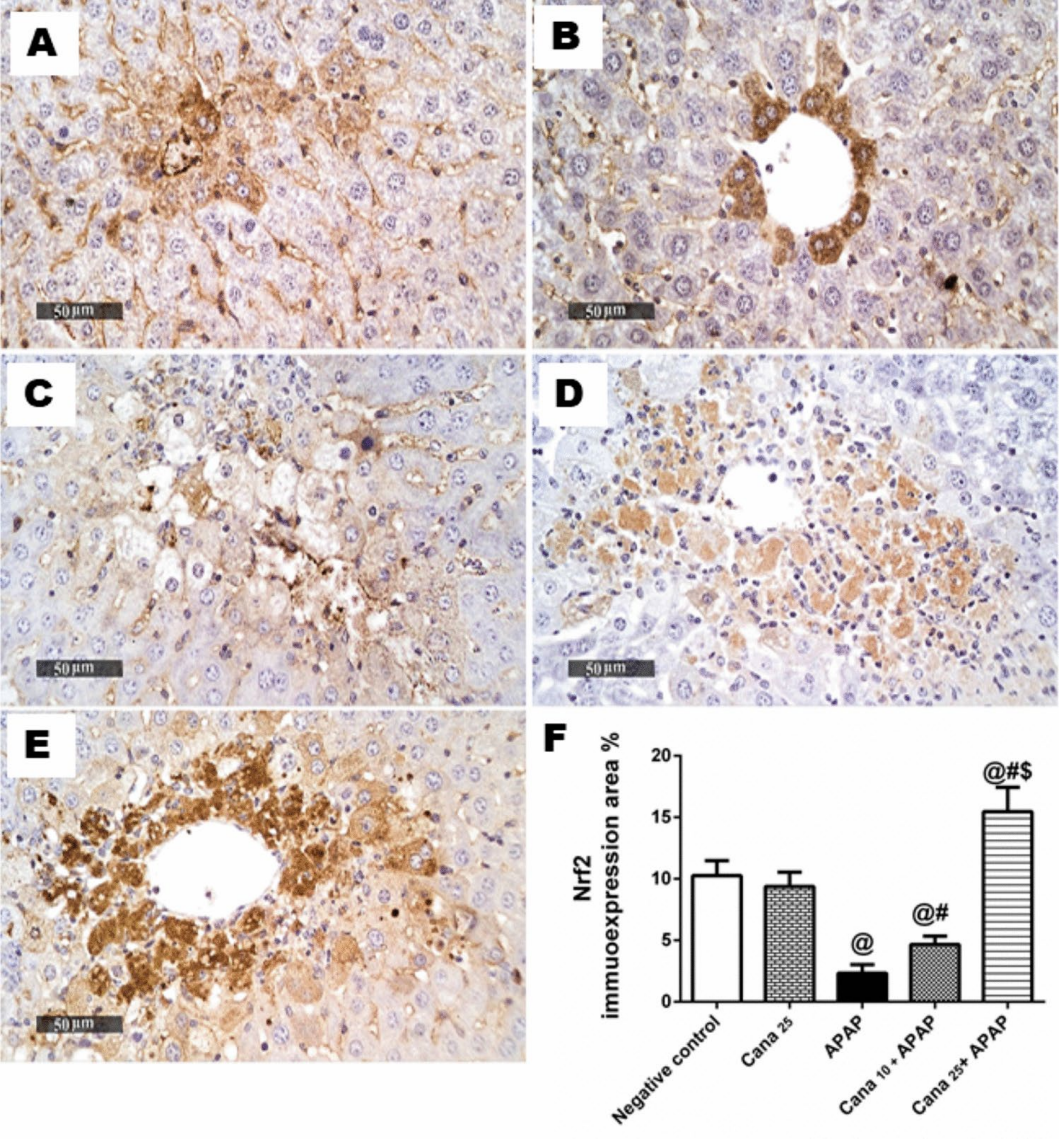
A representative micrograph of hepatic Nrf-2 immuno-expression. Section of **(C)** APAP shows a decrease in Nrf-2 expression compared to sections of **(A)** Negative control, and **(B)** Cana_25_.Section of **(D)** Cana_10_ and **(E)** Cana_25_ groups reveal an increase in Nrf-2 immuno-expression area, (scale bar = 50 μm). Panel **(F)** represents area % Nrf-2 immuno-expression, expressed as mean ± SD (n = 3) and data were analyzed using one-way ANOVA followed by Tukey’s *post-hoc* test (P < 0.05). As compared to (@) Negative control, (#) APAP, and ($) Cana_10_ + APAP groups. APAP: acetaminophen; Cana_10_: 10 mg/kg canagliflozin; Cana_25_: 25 mg/kg.

**Fig. 5:**
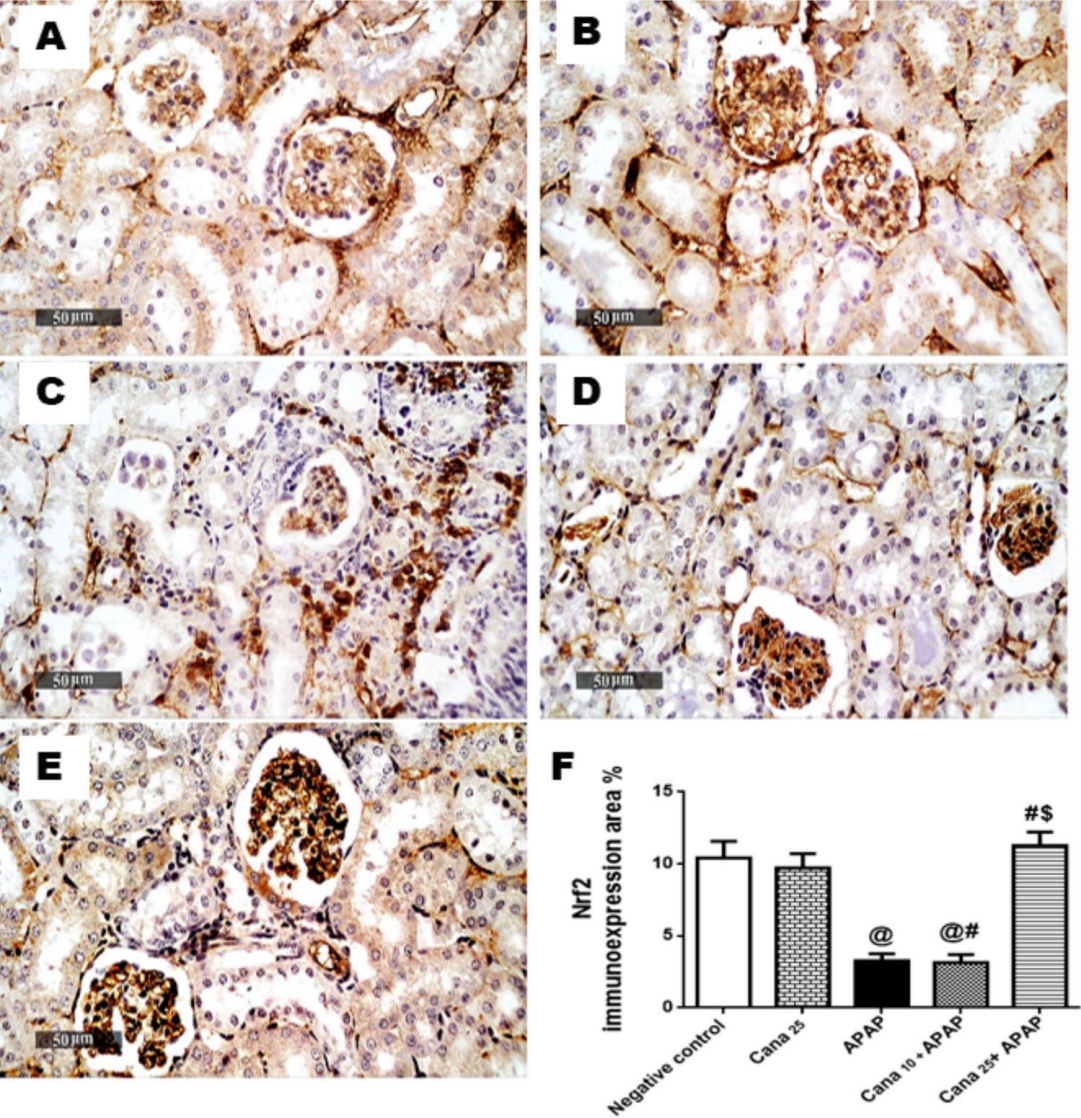
A** r**epresentative micrograph of renal Nrf-2 immuno-expression. Section of (C) APAP shows a decrease in Nrf-2 expression, compared to sections of **(A)** Negative control and **(B)** of Cana_25_. Section of **(D)** Cana_10_ and **(E)** Cana_25_ groups reveal an increase in Nrf-2 immuno-expression area, (scale bar = 50 μm). Panel **(F)** represents area % Nrf-2 immuno-expression, expressed as mean ± SD (n = 3), and data were analyzed using one-way ANOVA followed by Tukey’s *post-hoc* test (P < 0.05). As compared to the (@) Negative control, (#) APAP, and ($) Cana_10_ + APAP groups. APAP: acetaminophen; Cana_10_: canagliflozin10 mg/kg; Cana_25_: 25 mg/kg canagliflozin; nuclear factor-erythroid-2-related factor 2.

**Fig. 6:**
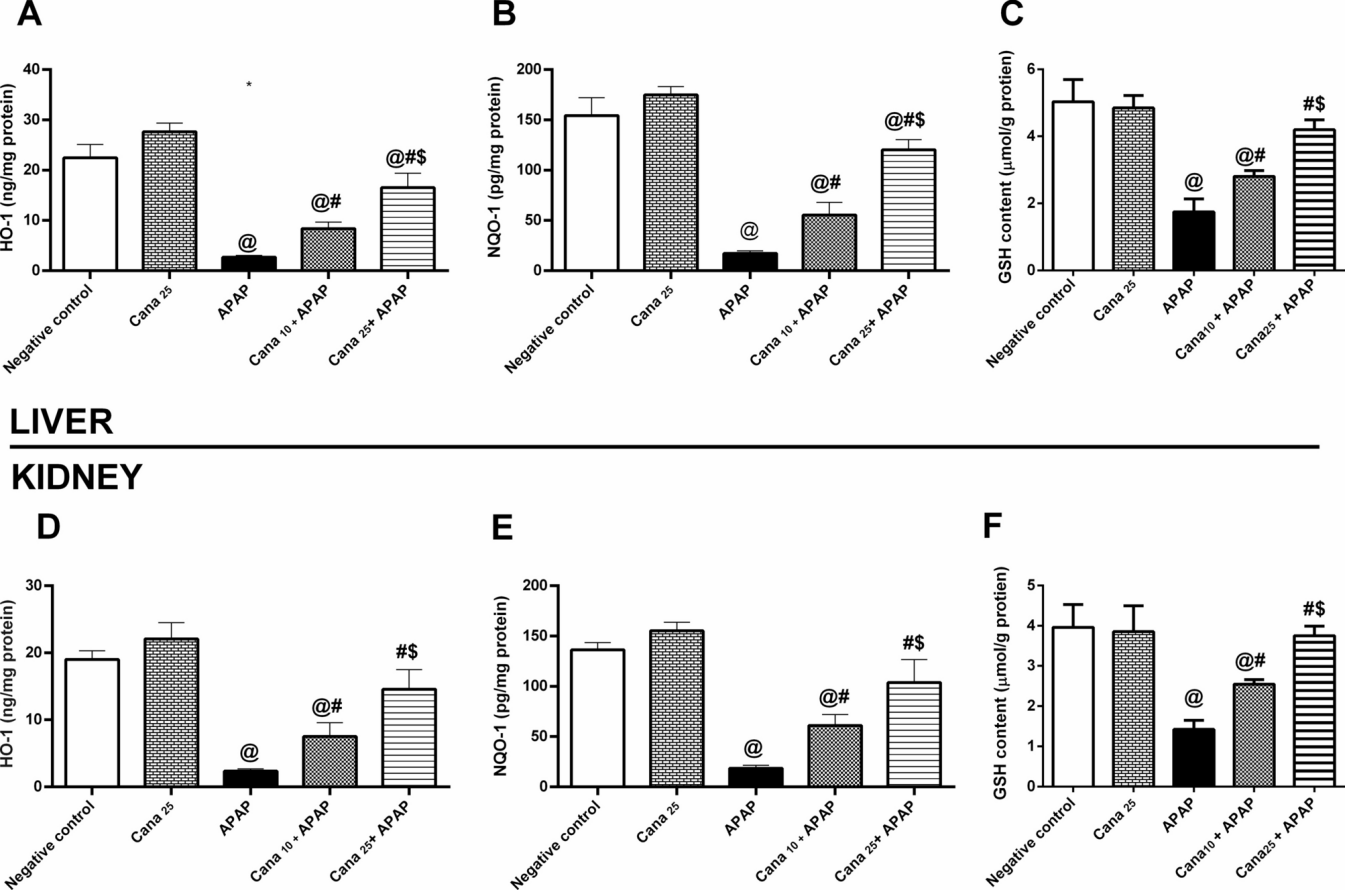
Effect of canagliflozin pre-treatment on hepatic and renal **(A, D)** HO-1 and **(B, E)** NQO-1, and **(C, F)** GSH contents on acetaminophen-treated mice. Data are presented as mean ± SD (n = 6) and were analyzed using one-way ANOVA followed by Tukey’s *post- hoc* test. P < 0.05, as compared to the (@) Negative control, (#) APAP, and ($) Cana_10_ + APAP groups. APAP: acetaminophen; Cana_10_: canagliflozin 10 mg/kg; Cana_25_: 25 mg/kg canagliflozin; Hemoxgenase-1; HO-1: GSH; glutathione: NQO-1; NAD(P)H quinone dehydrogenase 1.

Following inhibition of APAP to p-Akt/ p-GSK3β hub, the APAP insult evoked an attenuation in the hepato-renal antioxidant system. Nrf-2 immunohistochemistry sections of the APAP group of both hepatic (Fig. 4C) and renal (Fig. 5C) tissues revealed a marked dwindled Nrf-2 immuno-expression of 77% (Fig. 4F) and 69% (Fig. 5F), respectively, as compared to the negative control animals (Fig. 4A, 5A). Additionally, as presented in Fig. 6, the inhibitory effect of APAP on Nrf-2 entailed its downstream molecule in liver and kidney HO-1 (Fig. 6A, 6D) and NQO-1 (Fig. 6B, 6E) contents, in addition to depletion of GSH contents (Fig. 6C, 6F), respectively, relative to the negative control group. Interestingly, pre-treatment with Cana25 prior to APAP injection restored the antioxidant signal, as proven by the heightened area percent of Nrf-2 immno-expression in liver and kidney sections by 6.6 and 3.4-folds, respectively (Fig. 4E, 5E), and improved the decline contents of HO-1 (6-folds) (Fig. 6A, 6D), NQO-1 (6.9 and 3.5 folds) (Fig. 6B, 6E), and 15 GSH (2.4 and 2.6) (Fig. 6C, 6F), respectively, compared to APAP-treated mice. Moreover, Cana25 displayed a significant restoration of the Nrf-2/HO-1/NQO-1/HO-1 pathway compared to Cana10 in APAP-treated mice (Fig. 4D, 5D, 4F, 5F).

Given that the data collected from the Cana25 group (Fig. 4B, 5B) did not show any significant variation when compared to the negative control group, all comparisons across groups were made in relation to the negative control group.

### Canagliflozin modulates p-AMPK-α/SOCS-3 trajectory in APAP induced hepato-renal injury in mice

**Fig. 7:**
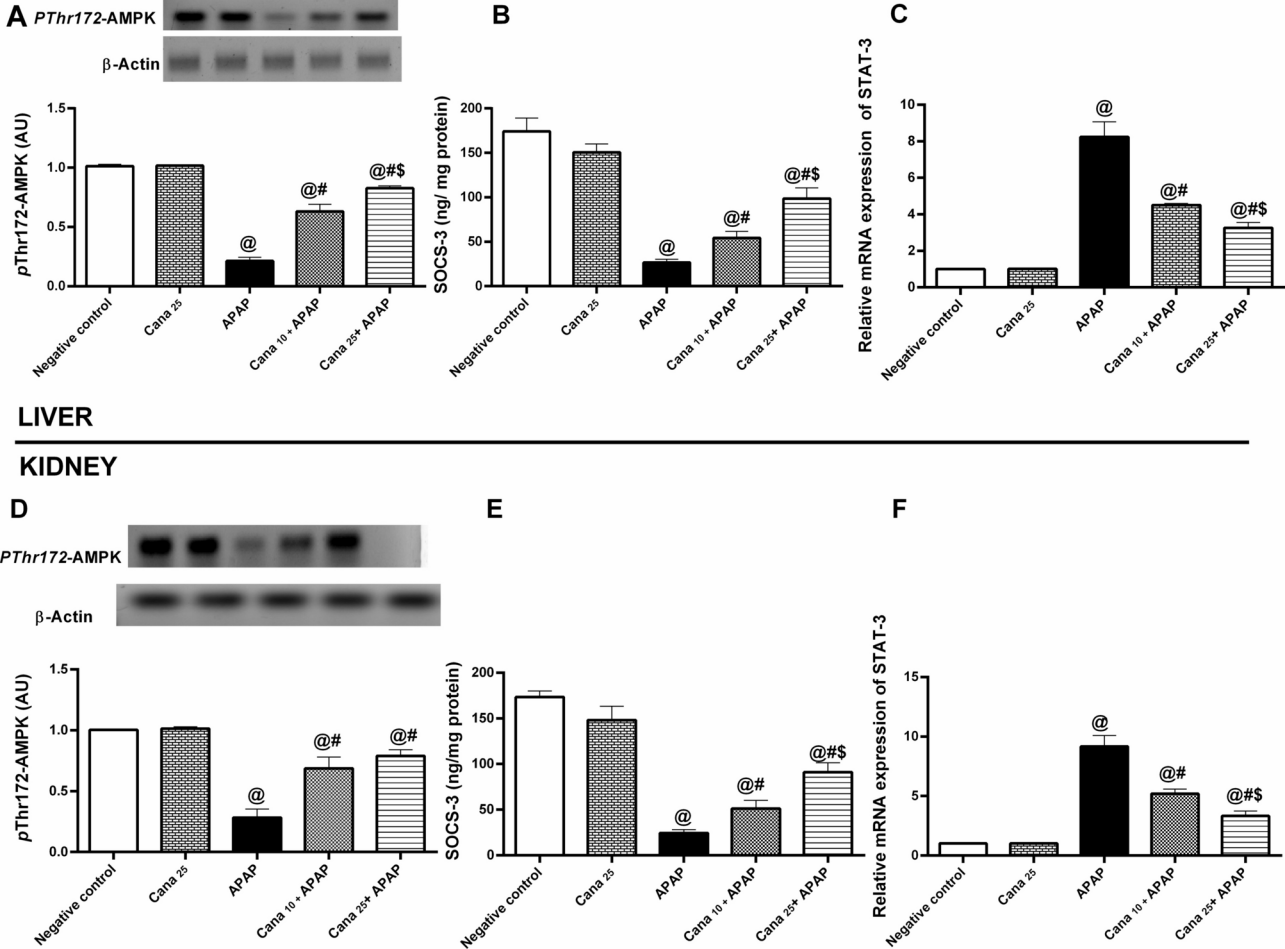
Effect of canagliflozin pre-treatment on hepatic/renal (**A**, **D**) *P*Thr172-AMPK protein expression, (**B**, **E**) STAT-3 gene expression, and (**C**, **F**) SOC-3 content on acetaminophen-treated mice. Data are presented as mean ± SD (n = 3–6) and were analyzed using one-way ANOVA followed by Tukey’s *post-hoc* test. P < 0.05, as compared to the (@) Negative control, (#) APAP, and ($) Cana_10_ + APAP groups. APAP: acetaminophen; *p-*AMPK-α*:* AMP-activated protein kinase phosphorylated at *Thr172;* AU: arbitrary unit; Cana_10_: 10 mg/kg canagliflozin; Cana_25_: 25 mg/kg canagliflozin; SOCS3: suppressor of cytokine signalling-3; STAT-3: signal transducer and activator transcription 3. Original blots are presented in supplementary Fig. 1.

In the APAP group, as illustrated in Fig. 7, liver samples showed a sharp drop in (A) *P*Thr172-AMPK-α protein expression by 79% and (B) SOCS-3 contents by (85%), but a significant upsurge in STAT-3 mRNA expression (8-folds) as compared to the normal group. The same APAP inhibitory effect on ***p*-**AMPK-α (72%) and SOC-3 (86%) with elevation of STAT-3 (9-folds), in kidney samples were obtained. Contrarily, pre-treated mice with Cana (10 and 25 mg/kg) elevated ***p*-**AMPK-α and suppressor of cytokine signalling-3 (SOC-3) with a subsequent reduction of STAT-3 compared to the APAP group, indicating a Cana anti-inflammatory effect, and again, Cana_25_ has a significant modulating effect in this signaling pathway relative to Cana_10_.

### Canagliflozin ameliorates APAP-provoked hepatic/renal histopathological alterations

**Fig. 8:**
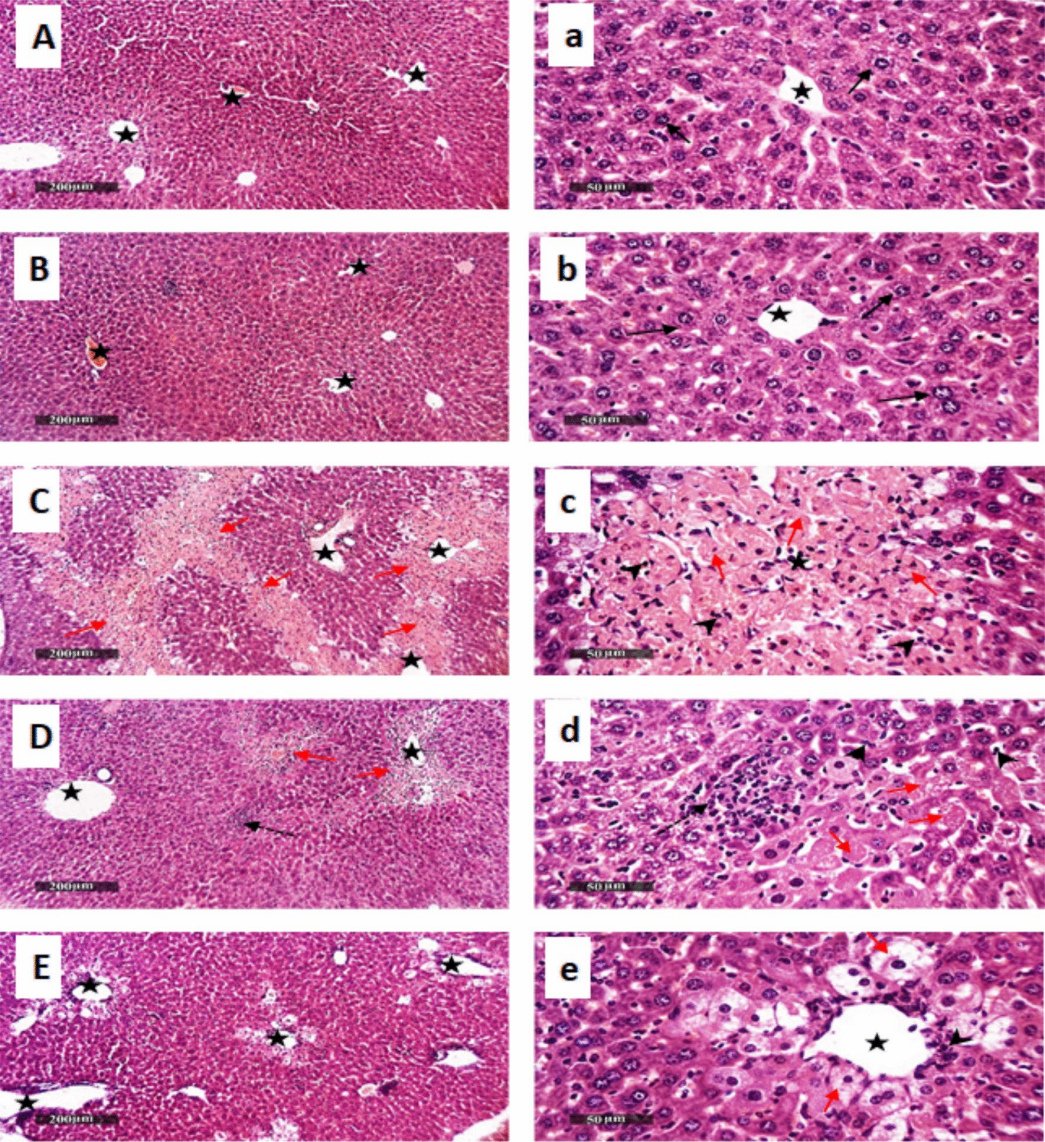
Descriptive photomicrographs of H&E staining presenting canagliflozin hepatoprotective effect in APAP-treated mice. The **(A, a)** Negative control and **(B, b)** Cana _25_ groups show apparent intact hepatocytes **(black arrow),** intact vasculatures **(star),** as well as hepatic sinusoids without abnormal cellular infiltrates and normal appearance, while those of **(C, c)** APAP group show pericentrally bridged hepatocellular necrosis **(red arrow),** inflammatory cell infiltrate **(arrow head),** and dilatation of hepatic vasculatures **(star).** However, **(D, d)** Cana _10_ + APAP and **(E, e)** Cana _25_ + APAP sections have prominent dissolution of hepatocellular bridging necrotic changes and records of apparent intact hepatocytes **(black arrow),** pericentral inflammatory cells infiltrates **(arrow head),** as well as ballooning degeneration with nuclear pyknosis of pericentral hepatocytes **(red arrow)** as well as dilatation of hepatic vasculatures **(star).** (Scale bar = 50 and 200 μm.). APAP: acetaminophen; Cana10: 10 mg/kg canagliflozin; Cana25: 25 mg/kg canagliflozin.

To further confirm the protective role of Cana in this model, we assessed the extent of liver and kidney alterations by hematoxylin–eosin staining. As indicated in Fig. 8, the liver sections of the (C, c) APAP group showed severe pericentral bridged hepatocellular necrosis with moderate records of inflammatory cell infiltrates accompanied by dilatation of hepatic vasculatures**,** a**s** compared to the normal liver architecture of normal samples (A, a, B, b). Contrariwise, sections of (E, e) Cana_25_ + APAP ameliorate (C, c) APAP-induced hepatic tissue damage with marked hepatoprotective efficacy as compared to (D, d) Cana_10_ + APAP revealed prominent dissolution of hepatocellular bridging necrotic changes and significant higher records of apparent intact hepatocytes accompanied by mild pericentral inflammatory cell infiltrates. But persistent mild ballooning degeneration with nuclear pyknosis of pericentral hepatocytes was shown as well as dilatation of hepatic vasculatures**.**

**Fig. 9:**
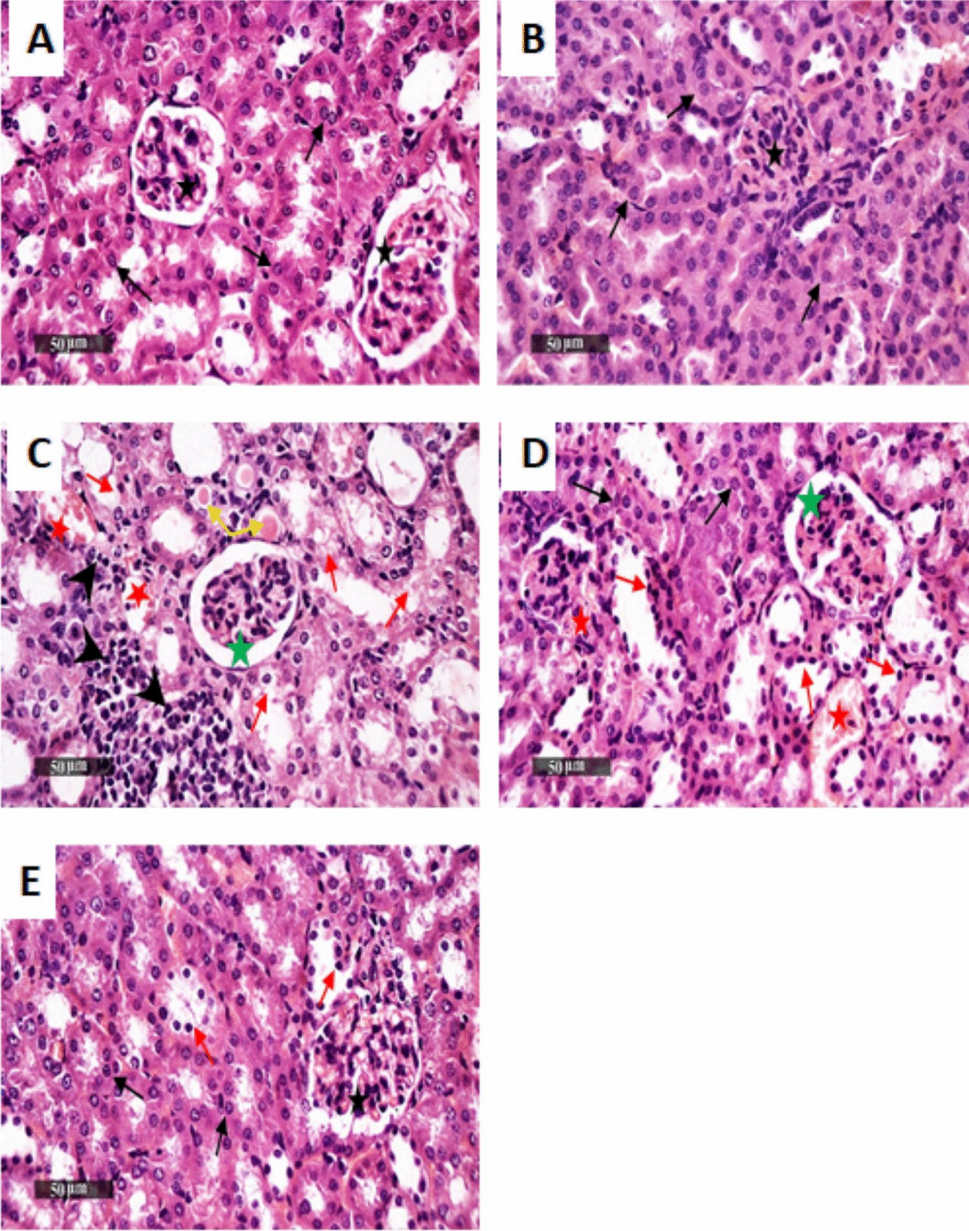
Descriptive photomicrographs of H&E staining present as canagliflozin renal protective effect in APAP-treated mice. Stained sections from the **(A)** negative control group and **(B)** Cana _25_ demonstrated normal histological structures of renal parenchyma with apparent intact renal corpuscles **(black star),** and renal tubular segments with almost intact tubular epithelium **(black arrow).** Whereas section of **(C)** shows tubular epithelial vacuolar degeneration and necrotic changes of different nephronal segments **(red arrow),** intraluminal eosinophilic casts **(yellow arrow),** along with tubular cystic dilatation and Bowman’s spaces dilatation **(green star),** as well as interstitial mononuclear inflammatory cell infiltrates **(arrow head)** and congested vasculatures **(red star)**. Section **(D)** Cana _10_ + APAP indicates records of degenerative changes of tubular epithelial cells **(red arrow)** alternated with a few apparent intact tubular segments **(black arrow)**, dilatation of Bowman’s spaces **(green star)** with congested vasculatures **(red star),** as well as obvious interstitial inflammatory cells infiltrates and intraluminal casts. Finally, section **(E)** Cana _25_ + APAP reveals intact, well-organized tubular segments ***(black arrow)*** with few records of tubular degenerative changes ***(red arrow)*** and almost intact renal corpuscles ***(star)*** with intact vasculatures. (Scale bar = 50 μm). APAP: acetaminophen; Cana_10_: 10 mg/kg canagliflozin; Cana_25_: 25 mg/kg canagliflozin.

In the same context, investigation of kidney sections revealed (C) APAP-induced prominent renal damage as shown in Fig. 9, which was displayed by marked diffuse tubular epithelial vacuolar degeneration and necrotic changes of different nephronal segments**,** intraluminal eosinophilic casts**,** and marked tubular cystic dilatation. In addition to a moderate dilatation of Bowman’s spaces**,** moderate interstitial mononuclear inflammatory cells infiltrate with congested vasculatures, indicating morphological changes compared to the normal renal architecture of the (A) negative control and (B) the Cana_25_ groups**.** Herein, (E) Cana_25_ combated the APAP-induced deleterious effect of the kidney with substantially higher protective efficacy relative to Cana_10_ on renal parenchyma, as revealed via abundant figures of apparent intact, well organized tubular segments and almost intact renal corpuscles with intact vasculature, as well as sporadic few records of tubular degenerative changes. These results suggest that APAP induced obvious liver and kidney damage and Cana (25 mg/kg) protected against histopathological hepato-renal injuries with a marked effect compared to Cana (10 mg/kg).

## Discussion

Acetaminophen (APAP), or paracetamol, is the most commonly used analgesic and antipyretic worldwide. At overdose, its reactive metabolite produces oxidative stress (OS), mitochondrial dysfunction and inflammation, ending with cellular damage. First of its kind, this research confirms one of Cana’s unique preventive roles against APAP-induced liver and kidney damage. Our suggested approach for Cana to reduce the negative impacts of APAP is to activate *p-*AMPK-α, together with its downstream components, *p*-Akt and *p*-GSK3β. The Nrf-2/HO-1/NQO-1/GSH cue was restored, and oxidative indicators were successfully suppressed by Cana. Its ability to raise SOC-3 contents and downregulate STAT-3 gene expression further dampened the inflammatory cascade.

Canagliflozin (Cana), besides being used to treat type 2 diabetes mellitus by inhibiting sodium-glucose co-transporter-2 (SGLT2), Plosker et al.^28^ have been shown to improve kidney function in diabetic nephropathic patients and liver function in type 2 diabetes patients^29,30^. Cana showed a mitigation effect on APAP-induced nephrotoxicity in the current investigation, as indicated by the reduction of well-known kidney injury parameters: KIM-1, NGAL, creatinine, and BUN levels. These findings are in analogy with Cana’s effective results in a cisplatin-induced nephrotoxicity study, as reported by Abdelrahman et al.^13^. In the same pattern, liver functions were improved in the current study with Cana pretreatment by diminishing ALT, AST, and bilirubin serum levels. The later results consolidate the previous findings of Cana in thioacetamide-induced liver injury^31^.

APAP-induced OS is instigated by its reactive metabolite NAPQI, which depletes cellular GSH, endorsing uncontrolled ROS production^32^. The APAP-treated group showed lower GSH contents in liver and kidney tissues, possibly due to GSH conjugation with NAPQI. Cana was able to hamper OS and showed higher levels of GSH; these results are consistent with Guvenc et al.^33^.

The initiated OS leads to the expression of antioxidant molecules such as Nrf2; however, continuous exposure to oxidative insults often confines Nrf2-induced protection and gives rise to great lipid peroxidation, which has a major deleterious consequence^32,34^. This observation aligns with the findings from the APAP-treated group. On the other hand, Nrf2 activation can promote the transcription of downstream detoxifying enzymes NADPH quinine oxidoreductase-1 (NQO-1) and heme oxygenase-1 (HO-1) that counteract OS and alleviate oxidative damage that happened at APAP toxicity^25^. Cumulating evidence has proposed that amplifying Nrf2 and its downstream molecules substantially diminished OS and inflammatory responses, thus improving hepatic and kidney injury induced by APAP^35–37^. Hence, the Nrf2 signaling pathway is considered an attractive target for reducing APAP-mediated toxicity^38^. This veracity has been documented herein in the Cana-treated group, suggesting its antioxidant effect, by enhancing Nrf-2/HO-1, NQO-1 and GSH compared to that in APAP-treated groups. This aligns with studies suggesting that Cana increases the expression of Nrf-2 and related antioxidant molecules, which offers protection in models of OS, such as isoprenaline-induced oxidative stress model^12^ and adenine-induced chronic kidney disease model^39^.

In order to obtain a deeper understanding of Cana’s underlying hepato-reno protective role against APAP-induced liver and kidney toxicities, our study specifically emphasized the role of AMP-activated protein kinase-α (*p*-AMPK-α) and its downstream cascade. During APAP overdose, *p*-AMPK-α, an intracellular energy sensor, is considered one of the markers of APAP-related damage^16^, and its activation ameliorates APAP-induced injury. Cai et al.^40^ reported that activation of *p-*AMPK-α ameliorates the consequences of OS. Recently, various literatures have focused on the importance of *p*-AMPK-α in kidney and liver problems, Viz., cisplatin-induced cytotoxicity^23^, thioacetamide-induced liver injury, and alcohol-induced fatty liver diseases^41,42^. Cana has been shown to exert protective effects via activation of *p*-AMPK-α. This was documented previously that Cana had the ability to overcome Carfilzomib toxicity through enhancement of *p*-AMPK-α^43^. These results showed Cana’s ability to modulate *p*-AMPK-α, as a key parameter, in mitigation renal and hepatic damage. from agents such as ASAP or other hepatotoxins.

One of the downstream molecules of *p*-AMPK-α activation are *p-*Akt/*p-*GSK3β, which are inhibited in liver- and kidney-treated APAP animals. *p*-AMPK-α was an upstream regulator for phosphorylation of Akt at the Ser473 residue^44,45^. Akt indorses survival by negatively regulating GSK3β via phosphorylation at the serine 9 residue, leading to inhibition of its activation^18,22,46^. Moreover, it has been reported that the cross-linking between these molecules gives cells a chance to protect against oxidative and inflammatory insults, as reported previously by Jin et al.^47^. Wang et al.^48^ stated that activation of the *p*-AMPK-α/*p*-Akt/*p*-GSK3β pathway is therapeutically effective against APAP-induced toxicity, which aligns with our findings that showed the deteriorating effect of APAP on the *p*-AMPK-α/*p*-Akt/*p*-GSK3β pathway. On the other hand, Cana exhibited a protective role in alleviating oxidative stress and tissue damage by modulating the *p*-AMPK-α/*p*-Akt* p*-GSK3β pathway, as documented previously against OS and inflammation in renal tissues under toxic stress induced compounds^12,49^. These findings are supported with our results where, *p*-AMPK-α/*p*-Akt* p*-GSK3β pathway had been recovered in Cana-treated groups after APAP- injury induction in both liver and kidney tissues.

In order to upregultes antioxidative effect, activated Akt with subsequent phosphorylation of GSK3β resulted in phosphorylation of Fyn-kinase leading to its inhibition. The reduction of Fyn-kinase facilitates Nrf-2 activation^50^. These outcomes supported with our results using Cana in both liver and kidney tissues by modulating p-Akt/p- GSK3β/Fyn-Kinase signalling pathway in improving Nrf-2 activity against APAP toxicity. Similarly, it has been reported that the inhibition of Fyn-kinase is considered a novel therapeutic target against kidney injury^51,52^. Our findings indicated that the Cana antioxidant impact might be related to inhibiting Fyn-kinase gene expression, a novel target molecule disclosed herein for interpreting the Cana antioxidant effect.

Inflammation is one of the key nodes in APAP-induced liver and kidney toxicity. Metabolites of APAP promote the release of inflammatory mediators that participate in deleterious effects on liver and kidney cells^53^. One of the important inflammatory signals that are activated is STAT-3, which is mediated via JAK proteins in response to cytokine binding^54^. Upon activation of STAT-3 signaling, its downstream molecule, the suppressor of cytokine signaling-3 (SOC-3), was negatively regulated and led to further inflammation^55^. On the contrary, SOC-3 activation plays an important role in reducing inflammation via inhibiting the cytokine signaling pathway and results in the inhibition of STAT-3 activation^6^. It has been reported that HO-1 negatively regulates the STAT-3 axis, which results in counteracting OS, inflammation, and apoptosis, accounting for tissue damage in the APAP model^6^. Cana herein attenuated STAT-3 and increased SOCS-3 in both renal and hepatic tissues, aligning with Sabe et al.^56^, who testified that Cana diminished JAK/STAT activation in chronic myocardial ischemia. The impact of Cana therapy on the STAT-3 pathway may be attributed to *p-*AMPK-α upregulation; this notion can be supported by Tsogbadrakh et al.^57^, who reported that *p-*AMPK-α played a role in STAT-3 suppression and SOCS-3 upregulation in cisplatin-induced acute kidney injury. Cana’s anti-inflammatory effect might also be attributed to the direct suppression of Fyn-kinase. Saminathan et al.^58^ earlier emphasized this, demonstrating that Fyn-kinase mediates inflammation in a mouse model of endotoxemia.

To summarize, our findings reveal that positive impact of Cana mitigates APAP-induced liver and kidney damage, primarily through activation *p-*AMPK-α. This activation regulates both the STAT-3/SOCS-3 axis and the p-Akt/*p*-GSK3β/Fyn-kinase/Nrf-2 pathway, reducing inflammation by suppressing STAT-3 and evoking SOCS-3 contents. Additionally, Cana’s antioxidant effect likely arises from the activation of the *p*-AMPK-α/p-Akt/p-GSK3β pathway, which facilitates the activation of Fyn-kinase/Nrf-2/HO-1/NQO-1/GSH pathway, counteracting oxidative stress caused by APAP overdose. Our research serves as a foundational step by offering valuable ground for future investigations, and further specific studies should focus on manipulating individual pathways to clarify the specific mechanism of Cana against ASAP toxicity.

## Supplementary Information

Supplementary Information 1.

Supplementary Information 2.

Supplementary Information 3.

Supplementary Information 4.

Supplementary Information 5.

Supplementary Information 6.

Supplementary Information 7.

Supplementary Information 8.

Supplementary Information 9.

Supplementary Information 10.

Supplementary Information 11.

Supplementary Information 12.

Supplementary Information 13.

Supplementary Information 14.

Supplementary Information 15.

Supplementary Information 16.

Supplementary Information 17.

Supplementary Information 18.

Supplementary Information 19.

Supplementary Information 20.

Supplementary Information 21.

## Data Availability

All data can be fully available upon reasonable request to the corresponding author.

## Abbreviations

APAP: Acetaminophen
Cana: Canagliflozin
GSK3β: Glycogen synthase kinase-3β
HO-1: Heme oxygenase 1
NOQ-1: NADPH quinone oxidoreductase-1
Nrf-2: Nuclear factor erythroid 2-related factor 2
*p*-Akt: Protein kinase B
SOCS-3: Suppressor of cytokine signaling-3
STAT-3: Signal transducer and activator transcription 3
