# Supplementary figures and images for: Canagliflozin alleviates acetaminophen-induced renal and hepatic injury in mice by modulating the p-GSK3β/Fyn-kinase/Nrf-2 and p-AMPK-α/STAT-3/SOCS-3 pathways
Source: Sci Rep. 2025 Jan 3;15:729. doi: 10.1038/s41598-024-82163-7 (PMC11699121; doi:10.1038/s41598-024-82163-7)

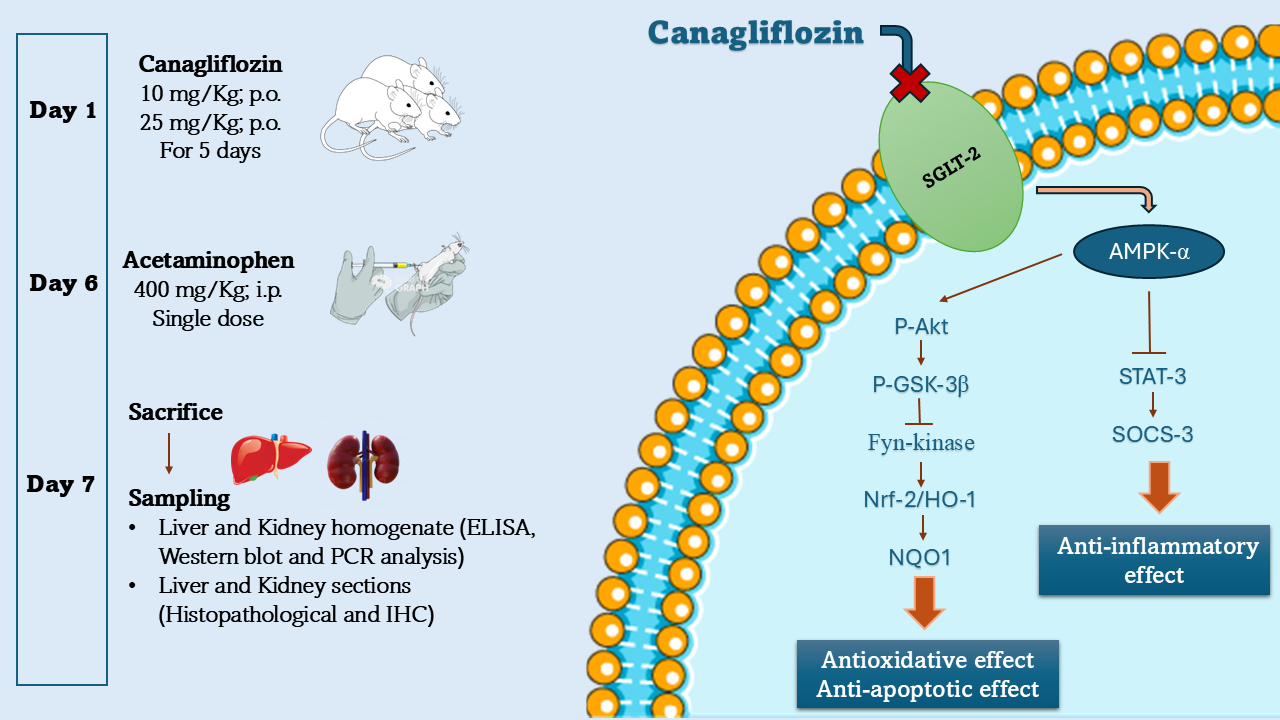

Supplement: Supplementary file 17 — Supplementary Information 17. [file 41598_2024_82163_MOESM17_ESM.tif]
